# Tailoring electronic and optical properties of hBN/InTe and hBN/GaTe heterostructures through biaxial strain engineering

**DOI:** 10.1038/s41598-024-51303-4

**Published:** 2024-01-11

**Authors:** A. Šolajić, J. Pešić

**Affiliations:** 1 Laboratory for 2D materials, Center for Solid State Physics and New Materials, Institute of Physics Belgrade, University of Belgrade, Pregrevica 118, 11080 Belgrade, Serbia; 2 Chair of Physics, Department Physics, Mechanics and Electrical Engineering, Montanuniversität Leoben, 8700 Leoben, Austria

**Keywords:** Electronic properties and materials, Two-dimensional materials, Electronic structure

## Abstract

In this research study, we systematically investigate the electronic and optical properties of van der Waals heterostructures (HSs) consisting of InTe (GaTe) and hBN monolayers, subjected to controlled biaxial strain. Our analysis demonstrates that the application of strain induces noteworthy alterations in the electronic band structure, enabling precise manipulation of the band gap and augmentation of the absorption properties of these structures. Employing density functional theory, we conduct a comprehensive examination of the influence of strain on the electronic and optical characteristics of these HSs. Our investigation showcases the remarkable potential of strain engineering in rendering these heterostructures into efficient and robust wide-range absorbers, particularly optimised for the visible spectrum, underscoring their relevance in various photonic and optoelectronic applications, paving the way for integration into advanced nanodevices.

## Introduction

Van der Waals (vdW) heterostructures (HSs), a class of materials composed of stacked two-dimensional materials held together by van der Waals forces, have attracted significant attention in recent years due to their unique electronic and optical properties^1–3^. A noteworthy advantage inherent to vdW HSs is their remarkable amenability to precise manipulation and customisation. The weak nature of vdW forces allows the layers to be easily separated and manipulated, allowing the creation of an unlimited number of different structures with precise control over the electronic, optical, and mechanical properties. This makes it possible to design and fabricate materials with specific desired characteristics, offering countless new possibilities for their applications in modern nanodevices^4^. With their high-performance electronic and optical properties, novel HSs can be used for transistors^5^, solar cells^6,7^, lithium ion batteries^8,9^, light emitting devices^10,11^, photodetectors^12–15^, various sensors^16–18^, and many more. Recent research has further illuminated their promising potential as candidates for quantum computing applications^19^.

Delving deeper into the mechanisms of manipulation for vdW HSs not only enhances their properties but also unlocks a wider array of possibilities for applications in devices that fulfil the growing demands of today’s market, such as sensors and switches. The modulation of electrical properties in such structures can be achieved in many ways, with the most common approaches being doping, strain application, or the use of external electric or magnetic field. The study of strain in 2D materials and vdW HSs represents a dynamically evolving research frontier, granting us an additional level of precision in controlling material properties^20–22^.

Group III–VI monochalcogenide-based HSs have emerged as focal points of research attention, offering substantial promise for a broad spectrum of cutting-edge device applications. They find utility as Schottky barriers, high-performance 2D and flexible electronics, sensors, photodetectors, and more. Materials within the class of two-dimensional III–VI monochalcogenides are known for their high electron mobility, broad absorption spectra, and favourable elastic properties. Notably, the single-layer InTe is predicted to exhibit exceptional thermoelectric performance due to its remarkably low thermal conductance, boasting the highest merit figure, $ZT = 2.03$ at 300 K, among the III–VI monochalcogenide family^23^. Recent study was discussing electronic transport and thermoelectric properties of doped InTe, showing that p-type InTe doped with Bi, Ag, Mn, Sn or Sb exhibits the enhanced thermoelectric performance, mainly induced by reduced thermal conductivity^24^. Additionally, the InTe monolayer demonstrates a broad absorption spectrum, covering the ultraviolet to visible regions, with an absorption coefficient of up to 10$^{-5}$ cm$^{-1}$. In most recent report, two-dimensional InTe was synthesized in large-scale samples, as centimeter-scale 2D films on SiO$_2$/Si substrates^25^. InTe also exists in tetragonal phase, and its electronic band structure has a highly anisotropic character^26^ , marking it of high interest for electronic and thermoelectric applications. In that phase, structure has quasi-one-dimensional form, where one-dimensional In$^{1+}$ chains are observed, and additionally, the presence of In$^{1+}$ induces a localized gap state, responsible for the high intrinsic p-type doping of InTe^27^. Similarly to InTe , the monolayer GaTe exhibits comparable absorbing properties^28^, along with excellent UV light absorption. In the realm of single-layer materials, each member of this material family exhibits remarkable and distinctive properties. Nonetheless, single layer III–VI monochalcogenides possess one noteworthy limitation in their pristine form. The majority of these materials display sensitivity to oxidation upon exposure to ambient air^29–31^, especially in their single layer form, and need adequate material for passivisation and mechanical protection. Non-reactive with most chemicals, stable in air and resistant to oxidation, hBN is already known as an effective coating material in the form of thin films or monolayers^32–34^, and has been demonstrated successful for protection against oxidation and even for improving the electronic and optical properties of few-layered InSe and GaSe^35^.

Motivated by these results, in our previous work, we designed and investigated two novel heterostructures, hBN/InTe and hBN/GaTe, as detailed in^36^. These heterostructures displayed favourable electronic properties and an excellent broad absorption spectrum, enhanced and protected by the hBN layer, making them promising candidates for applications in photodetectors or field-effect transistors. Given the experimentally favourable binding energies and stacking versatility of both hBN/InTe and hBN/GaTe heterostructures, this study further delves into the intriguing potential of fine-tuning their properties through the application of controlled strain. In the following sections, we present a computational study of hBN/InTe and hBN/InTe under biaxial strain. The application of uniform biaxial strain does not alter the symmetry of the structure; instead, it allows us to tune the band structure and optical absorption.

## Computational methods

Calculations were carried out using density functional theory (DFT), as implemented in the Quantum Espresso (QE) software package^37^, based on plane waves and pseudopotentials. In all calculations, the Perdew–Burke–Ernzerhof (PBE) exchange correlation functional^38^ is used, along with norm-conserving pseudopotentials. The energy cutoff of 80 Ry was set for both structures after the convergence tests.

The Monkhorst pack of $16\times 16\times 1$ mesh for k-point sampling is used in geometric optimisation, total energy, and phonon calculations. For calculations of p-DOS and optical properties, a refined $64\times 64\times 1$ mesh is used. The band structure is calculated on 440 k-points along $\mathrm {\Gamma }$-M-K-$\mathrm {\Gamma }$ direction. To simulate the 2D structure, a vacuum of 20 $\mathrm {\mathring{A}}$ was added along the z-direction to avoid interactions between the layers. Geometry optimisation of the positions of the atoms and the lattice parameters is performed using the BFGS algorithm, with criteria for the maximum forces allowed between atoms of $10^{-6} \,\mathrm {Ry/\mathring{A}}$. To properly account for van der Waals force effects, the Grimme-D2 correction^39,40^ was included to obtain more accurate lattice constants and forces. The optical properties were calculated using the epsilon.x code in QE software, based on the random phase approximation (RPA).

## Results and discussion

**Figure 1:**
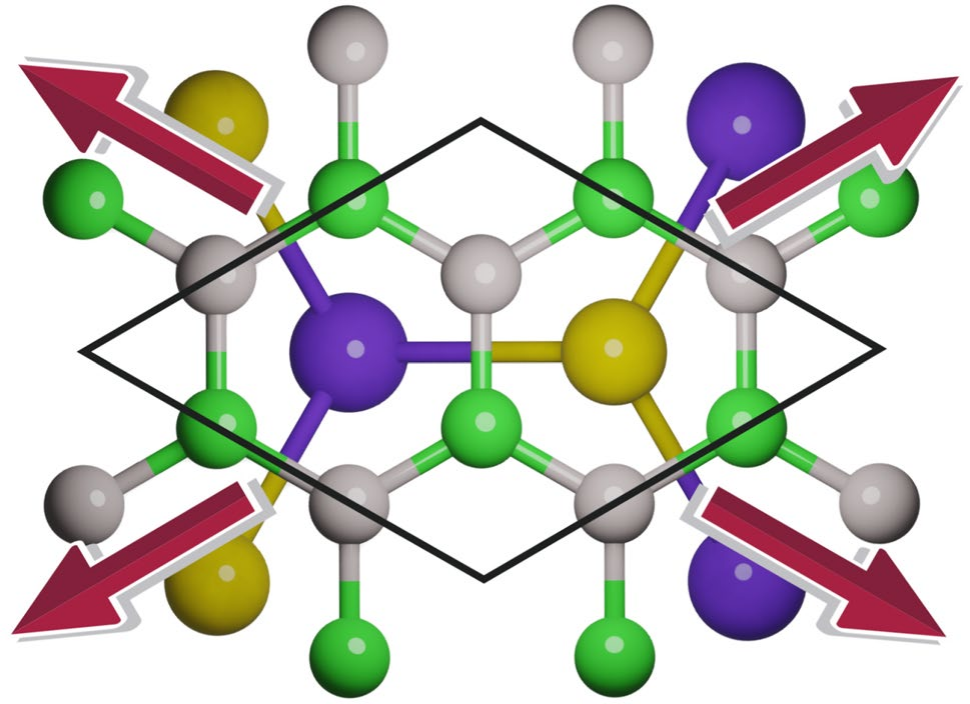
Top view of heterostructures, with arrows showing the tensile biaxial strain. B and N atoms are represented with a grey and green colour, In/Ga and Te atoms are coloured purple and yellow.

**Figure 2:**
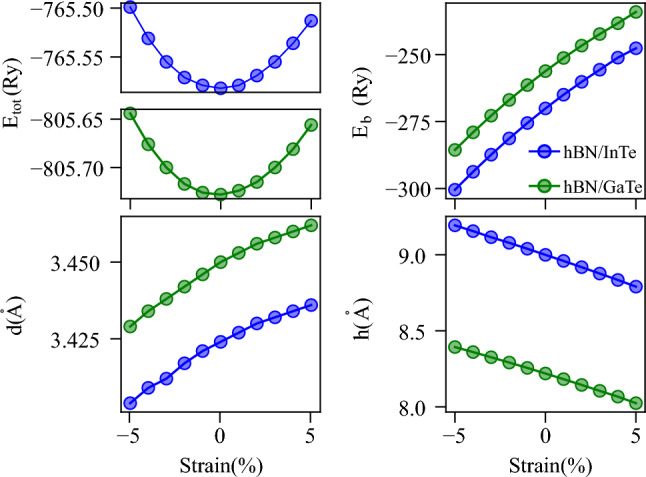
Dependence of total energy $E_{tot}$, binding energy $E_b$. distance between the layers *d* and the thickness of HSs *h* on applied strain. The blue lines and markers correspond to hBN/InTe, and green lines and markers correspond to hBN/GaTe.

As discussed in our previous work^36^, the unit cells of InTe and GaTe monolayer are $a = 4.371 \,\mathrm {\mathring{A}}$ and $a = 4.048 \,\mathrm {\mathring{A}}$, respectively. The unit cell of hBN is $a = 2.515 \,\mathrm {\mathring{A}}$, and for both structures, constructing the heterostructure consisting of a $\sqrt{3}\times \sqrt{3}$ supercell of hBN rotated for 30$\circ$ on top of $1\times 1$ unit cell of InTe or GaTe provides an excellent match. After complete optimisation of the lattice parameters and atom positions within the unit cell, the obtained unit cell is hexagonal, with a lattice constant of $a = 4.336 \,\mathrm {\mathring{A}}$ for hBN/InTe and $a=4.309 \,\mathrm {\mathring{A}}$ for hBN/GaTe. Resulting unit cell of hBN/InTe has induced strain of  0.8% on InTe layer and  0.3% on hBN layer, showing that HS constructed as described is almost an ideal match. The resulting unit cell of hBN/GaTe induces strain of  6% on GaTe and  0.8% on hBN which is slightly less perfect than hBN/InTe HS, but still agreeable. The top view of the structures is presented in Fig. 1. With all three types of stacking being energetically close to each other, we consider H-top stacking (the In(Ga) atom above the centre of the hBN hexagon), which has slightly lower total energy and binding energy compared to the B-top and N-top types. All constituting materials (hBN, InTe, GaTe) exhibit outstanding mechanical properties, demonstrating the ability to withstand significant biaxial strain strengths of more than 10%^23,28,41^ . From an experimental standpoint, achieving a controllable and precise strain beyond a few percent in complex structures like van der Waals (vdW) heterostructures is often challenging, if not impossible. Taking this into account, we chose to set the maximum absolute strain values at 5% even though both systems theoretically possess the capacity for higher strain tolerance. The uniform biaxial strain of − 5 to 5% is applied to both structures, with the step of 1%, and the geometry optimisation of the positions of the atoms within the unit cell is performed for all strained structures. The binding energies $E_b$ are calculated for all structures as follows:

1 $$\begin{aligned} E_b = E_{heterostr.} - E_{In(Ga)Te} - E_{hBN}, \end{aligned}$$

where $E_{heterostr.}$, $E_{In(Ga)Te}$ and $E_{hBN}$ represent the total energy of hBN/In(Ga)Te heterostructure, InTe or GaTe monolayer and hBN monolayer, respectively. Total and binding energies, $E_{tot}$ and $E_b$, along with the distance between the hBN and In(Ga)Te layers *d* and the width of the layer *h* are given in Table in the Supplementary file. First, the binding energies are negative in all cases, suggesting that all strained structures are experimentally feasible. It can be seen from and Fig. 2 that the total energy is lowest for HS without strain and increases exponentially with both positive and negative strains, as expected, since the initial system without strain was fully relaxed with respect to the lattice constants and atom positions within the unit cell. The distance between layers and the bond lengths change with different values of applied strain. Although the bonds in the InTe (GaTe) layer are slightly shortened when compressive strain is applied (mainly the In(Ga)–Te bonds), a significant difference is present in the angle of the In(Ga)–Te bond with respect to the horizontal plane, so the In(Ga)Te layer width is increased, and the inner Te atoms are positioned slightly closer to the hBN layer. In the case of tensile strain, the In(Ga)Te bonds are stretched, causing the layer width to decrease and the distance between the inner Te atoms and the hBN layer to increase. The complete data are given in Table 1 in the Supplementary file.

Before the introduction of strain into any structure, it is essential to conduct a thorough analysis of its mechanical characteristics. We used the Thermo pw code^42^ to compute the elastic constants. The code calculates the non-zero components of the stress tensor for a set of strains and obtains the elastic constants from the first derivative of the stress with respect to the strain. In this way, we can gain a solid basis to understand the relationship between strain and the mechanical reaction of the structure. For 2D systems, nonzero elastic constants follows the Hooke’s law under plane stress conditions:

2 $$\begin{aligned} \left[ \begin{array}{l} \sigma _1 \\ \sigma _2 \\ \sigma _3 \end{array}\right] =\left| \begin{array}{lll} c_{11} &{} c_{12} &{} 0 \\ 0 &{} c_{22} &{} 0 \\ 0 &{} 0 &{} c_{66} \end{array}\right| \cdot \left[ \begin{array}{l} \varepsilon _1 \\ \varepsilon _2 \\ \varepsilon _3 \end{array}\right] . \end{aligned}$$

For a hexagonal lattice, $C_{11}=C_{22}$ and $C_{12}=C_{21}$, $C_{66}=(C_{11}-C_{22})/2$, so there are only two independent elastic constants. In that case, Young’s modulus, Poisson’s ratio, and shear modulus are obtained from the following relations:

3 $$\begin{aligned} E_{Y}=C_{11} - \frac{C_{12}^2}{C_{11}}, \,\, \nu _{xy}=\frac{C_{12}}{C_{11}}, \,\, G_{xy}=C_{66}. \end{aligned}$$

For hexagonal 2D systems, layer modulus that represents the resistance of a sheet to stretching is calculated by following^43^:

4 $$\begin{aligned} \gamma ^{2D}=\frac{C_{11}+C_{12}}{2}. \end{aligned}$$

Calculated elastic constants and moduli are given in Table 1. Obtained elastic constants of single layer InTe and GaTe are in range of other III–VI monochalcogenides^44^. Constants $C_{11}=45.36$ N/m and $C_{12}=11.76$ N/m for InTe are close to constants of single layer InSe, and $C_{11}=65.62$ N/m and $C_{12}=15.30$ N/m for GaTe close to results for single layer GaSe. We calculated the elastic constants of hBN in order to validate our results, and obtained values are in agreement with the literature^43,45^. When the HSs are formed, all constants and moduli are significantly increased. Elastic constants $C_{11}$ and $C_{12}$ for hBN/InTe are $C_{11}=338.3$ N/m and $C_{12}=72.08$ N/m; $C_{11}=340$ N/m and $C_{12}=75.48$ for hBN/GaTe, and their values are roughly similar to the sum of individual constants of each layer in the heterostructure. Young modulus is increased to  323 N/m in HSs, which are high almost as in graphene (342–366 N/m for Young modulus and 206–212 N/m for layer modulus, according to the literature^43,46^), indicating high resistance to unidirectional compression as well to stretching. The results suggest that the presence of the hBN layer in our heterostructures not only shields the delicate monochalcogenide layers from oxidation but also provides effective mechanical protection, at the same time rendering the system more robust and resistant to deformations.

**Table 1:** Elastic constants, Young modulus, Poisson ratio, shear modulus and layer modulus for HSs and pristine InTe and GaTe monolayers.

1	InTe	GaTe	hBN	hBN/InTe	hBN/GaTe
$C_{11}$	45.36	65.62	290.77	338.30	340.00
$C_{12}$	11.76	15.30	63.93	72.08	75.48
$C_{66}$	16.52	25.16	113.42	132.94	132.26
$E_y$	42.31	62.05	276.41	322.94	323.24
$\nu _{xy}$	0.26	0.23	0.22	0.21	0.22
$G_{xy}$	16.52	25.16	113.42	132.94	132.26
$\gamma ^{2D}$	28.56	40.46	177.35	205.19	207.74

Units are given in N/m.

**Figure 3:**
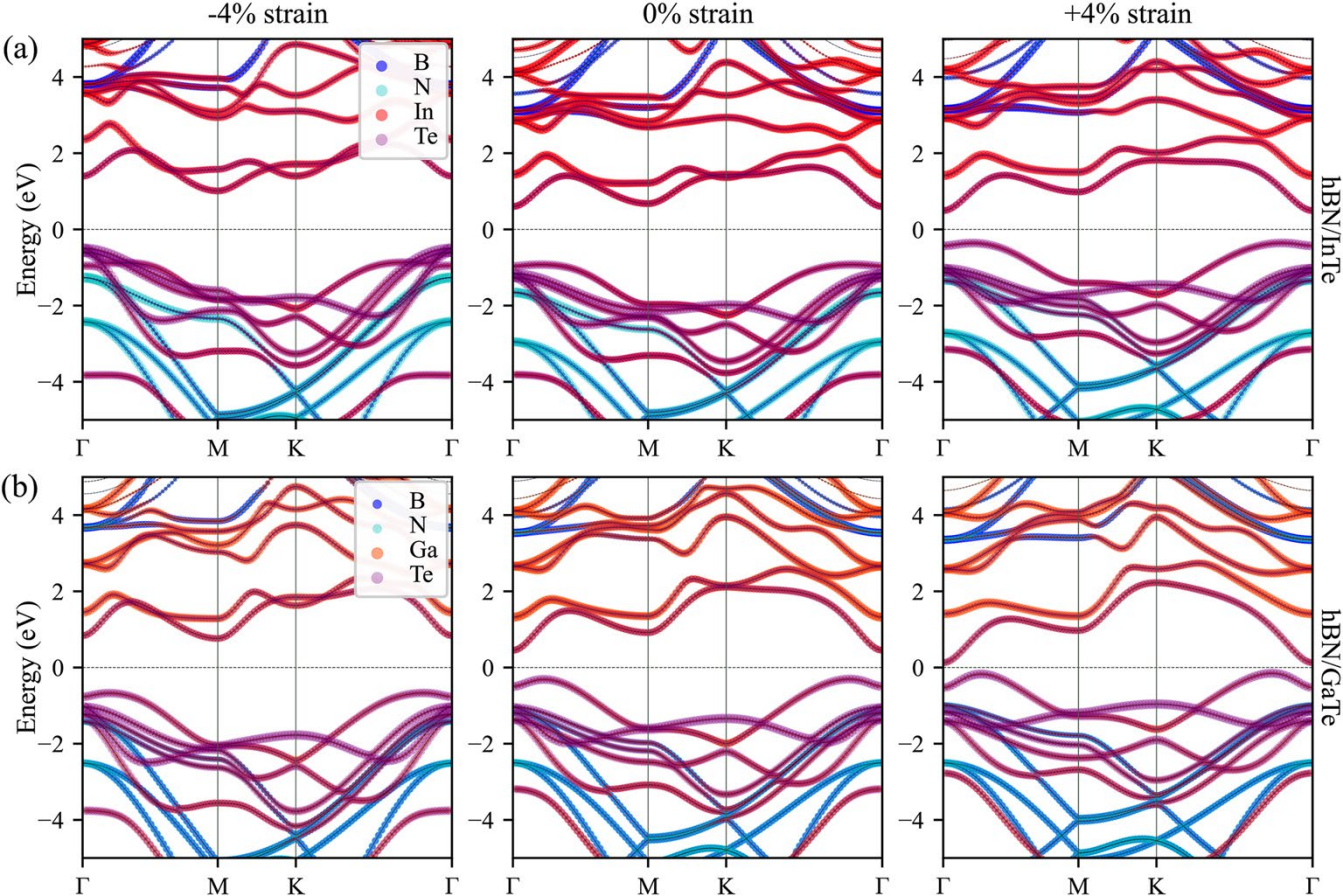
Band structure of (**a**) hBN/InTe HS and (**b**) hBN/GaTe. The width of the lines is proportional to the contribution of different atoms/states. The line width is proportional to the magnitude of projections of wavefunctions over atomic orbitals. The contributions from different atomic orbitals are presented in different colours, as shown in the legend.

In our investigation, we computed the band structures of both heterostructures (HSs) under various compressive and tensile strain conditions, building upon the foundation laid out in our previous study^36^. The band structures for HSs under − 4%, 0% and 4% strain are visually represented in Fig. 3. The band structures for all values of strain, from − 5 to 5%, are represented in Figures S1 and S2 in supplementary file. In their pristine, unstrained states, hBN/InTe and hBN/GaTe exhibit band gaps of $E_g = 1.53$ eV and $E_g = 0.76$ eV, respectively. Notably, both HSs display an indirect band gap configuration, with the valence band maximum (VBM) positioned near the $\Gamma$ point and the conduction band minimum (CBM) precisely at the $\Gamma$ point.

When the application of strain is introduced, significant alterations occur in both the band gap and the shape of the bands within the heterostructures. In the case of the hBN/GaTe heterostructure subjected to compressive strain, the band gap widens as the strain magnitude increases, reaching a maximum value of $E_g=1.49$ eV at $-5\%$ strain. Importantly, the valence band retains its shape while shifting downward to lower energy levels. In contrast, the conduction band exhibits distinct behaviour: the $\Gamma$-valley expands, whereas the energy level of the M-valley remains relatively stable. This results in both valleys having nearly identical energies at $-4\%$ strain. Conversely, the introduction of tensile strain leads to a contrasting effect. The upper region of the valence band near the $\Gamma$ point shifts upward, while the lower region of the conduction band at the $\Gamma$ point experiences a substantial descent. Consequently, at + 5% strain, the band gap narrows to only 0.24 eV.

The relationship between band gap and strain is systematically explored and plotted in Fig. 6. Remarkably, across the entire range of applied strains, from $-5$ to + 5%, the dependence closely approximates a linear decrease.

Turning our attention to the hBN/InTe heterostructure, we observe similar trends in band gap modulation under strain. However, a significant disparity arises in the conduction band behaviour, particularly under compressive strain. Here, the valence band also shifts downward as compressive strain intensifies, eventually falling below the group of bands situated around $-0.5$ eV at the $\Gamma$ point. Initially, in the relaxed state, the conduction band minimum (CBM) resides at the $\Gamma$ point, while the bottom of the M valley maintains nearly the same energy. However, as compressive strain is applied, the $\Gamma$-valley remains relatively stable in terms of energy, while the M-valley experiences a downward shift. Consequently, the CBM transitions from the $\Gamma$ to the M-point as strain surpasses $-1$%. This intricate interplay between the valence and conduction bands results in a band gap expansion, peaking at $E_g=1.69$ eV at $-2$% strain before gradually reducing to 1.39 eV at $-5$% strain.

In the case of tensile strain, the behaviour mirrors that of the hBN/GaTe heterostructure. The valence band shifts upward, and the CBM descends with increasing tensile strain, leading to a reduced band gap, which ultimately reaches a minimum of 0.70 eV at + 5% strain.

**Figure 4:**
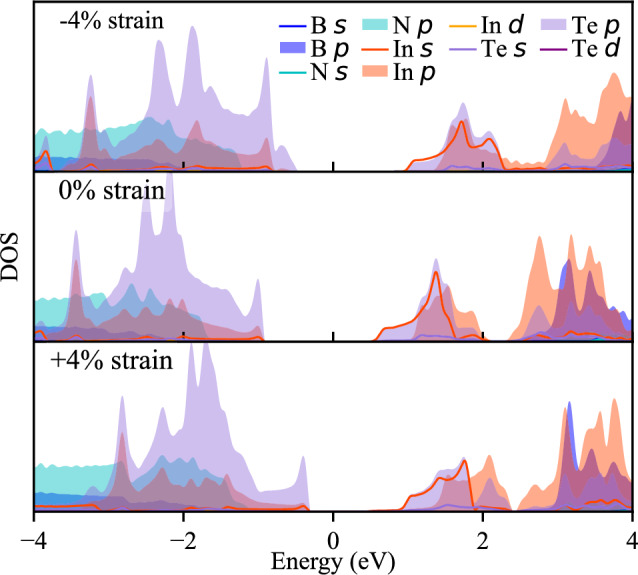
Projected density of states of hBN/InTe HS. Contributions from different atoms and states are represented with colours as in legend.

**Figure 5:**
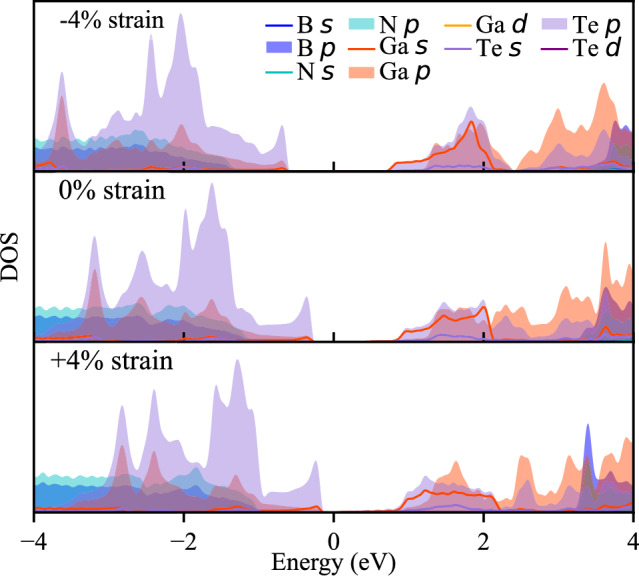
Projected density of states of hBN/GaTe HS. Contributions from different atoms and states are represented with colours as in legend.

The shifts in the position of valleys within the conduction band in response to varying strain values are not uncommon and have been observed in InTe and GaTe monolayers subjected to biaxial strain^28,47,48^. Furthermore, our analysis of the density of states (DOS) reveals that the bands closest to the Fermi level predominantly arise from the In(Ga) and Te states. DOS for HSs with − 4%, 0% and 4% strain are presented in Figs. 4 and 5 . Complete data with DOS for all strain values are shown in Figure S3 in supplementary file. The uppermost bands below the Fermi level primarily originate from the Te states, while the bands above comprise a combination of In(Ga) and Te states. Consequently, it can be inferred that the changes in the conduction band shape predominantly result from the influence of strain on the InTe(GaTe) layer itself, rather than being an inherent characteristic of the formed heterostructure. These findings underscore the profound influence of strain engineering on the electronic properties of these heterostructures, offering a promising avenue for precise control and modulation of their behaviour for tailored optoelectronic applications.

**Figure 6:**
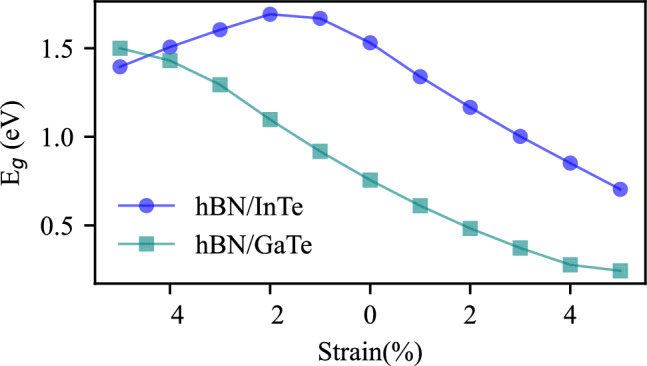
Band gap of hBN/InTe (indicated as blue line and circles) and hBN/GaTe (indicated as green line and squares) as a function of applied strain.

Significant variations in both the band gap and the shape of the bands can drastically alter dielectric function and absorption. The complex dielectric function $\epsilon (\omega )+ \epsilon _R(\omega )+i\epsilon _I(\omega )$ is obtained from DFT calculations in the RPA framework. The absorption coefficient $\alpha (\omega )$ is obtained directly from the dielectric function as follows:

5 $$\begin{aligned} \alpha (\omega ) = \sqrt{2}\frac{\omega }{c}\sqrt{\sqrt{\epsilon _R^2(\omega )+\epsilon _I^2(\omega )}-\epsilon _I(\omega )}. \end{aligned}$$

**Figure 7:**
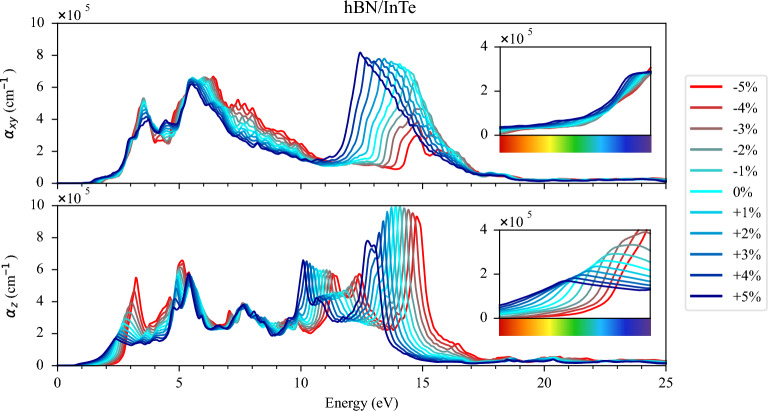
Absorption function of hBN/InTe HS for (**a**) in-plane ($\alpha _{xy}$) and (**b**) out-of-plane ($\alpha _{z}$) polarisations. Each colour represents a different value of the induced strain, from red (− 5% of strain) to blue (+ 5% of strain). The visible part of the spectrum is enlarged in the inset.

**Figure 8:**
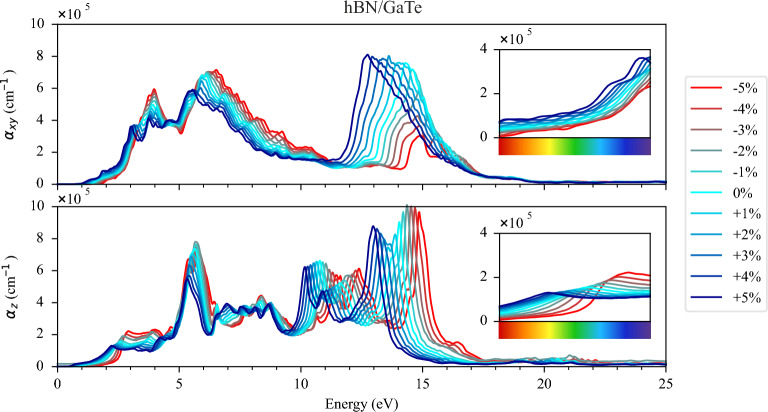
Absorption function of hBN/GaTe HS for (**a**) in-plane ($\alpha _{xy}$) and (**b**) out-of-plane ($\alpha _{z}$) polarisations. Each colour represents a different value of the induced strain, from red (− 5% of strain) to blue (+ 5% of strain). The visible part of the spectrum is enlarged in the inset.

Calculation of optical properties were performed within RPA. Optical properties of group III monochalcogenide members and similar structures as well of hBN were previously studied both with GW and RPA based methods, and it is shown that RPA can provide reasonably good results of dielectric function and its qualitative description^49–53^. In^54^, heterostructure of InSe with silicene, germanene and antimonene, imaginary part of the dielectric function calculated both with RPA over GGA and GW, and main difference observed is the shift of the dielectric function for 0.5–1 eV due to larger calculated band gap. The absorption coefficients of both HSs under different strain strengths are presented in Figs. 7 and  8, respectively. The band gap width variations induced by strain strength exhibit a notable influence on the absorption properties of both HSs. Specifically, compressive strain causes the absorption function to shift towards higher energies, while tensile strain results in a shift towards lower energies, a behaviour consistently observed in both HSs. However, the most striking disparity in absorption behaviour occurs in hBN/InTe, where the application of compressive strain induces the formation of a pronounced peak at approximately 3 eV for z-polarisation. This striking alteration is attributed to the significant reduction of the M-valley in hBN/InTe and is absent in hBN/GaTe under similar strain conditions. This study highlights the intricate interplay between strain and the optical properties of these heterostructures. The observed differences in absorption behaviour underscore the nuanced effects that strain engineering can exert, offering a pathway towards fine-tuning the optical characteristics of these materials for tailored optoelectronic applications.

## Conclusions

In this study, we systematically investigated the impact of biaxial strain on recently designed HSs composed of InTe (GaTe) and hBN monolayers using DFT. All the considered strained structures were found to be experimentally feasible, characterised by negative binding energies. Our band structure analysis revealed that strain offers a powerful tool for the precise manipulation of the band gap in these structures.

In the case of hBN/GaTe, we observed an almost linear relationship between band gap and strain, with band gap values increasing under compressive strain and decreasing under tensile strain. Specifically, the largest band gap of $E_g=1.49$ eV was achieved at − 5% strain, while it reduced to 0.24 eV at + 5% strain. In hBN/InTe, the manipulation of strain led to a decrease in the M-valley’s energy, effectively positioning it below the $\Gamma$-valley. This resulted in a band gap that decreased under strain stronger than − 2%, with band gap energies falling within the range of 0.70–1.69 eV.

Additionally, we examined the optical properties by calculating the dielectric functions and found that tensile strain substantially enhanced absorption in the low-energy spectrum, particularly in the visible spectrum. On the contrary, compressive strain increased absorption at 3 eV, but shifted the absorption function towards higher energies.

Our findings underscore the pivotal role of strain engineering in these HSs, offering precise control over their electronic and optical properties. Furthermore, these tunable properties open up possibilities for their utilisation in various sensor and switch applications.

### Supplementary Information

Supplementary Information.

## Data Availability

The datasets used and/or analysed during the current study are available from the corresponding author on reasonable request.
