# Supplementary material for: Tailoring electronic and optical properties of hBN/InTe and hBN/GaTe heterostructures through biaxial strain engineering
Source: Sci Rep. 2024 Jan 11;14:1081. doi: 10.1038/s41598-024-51303-4 (PMC10784494; doi:10.1038/s41598-024-51303-4)
Supplement: Supplementary file 1 — Supplementary Information. [file 41598_2024_51303_MOESM1_ESM.pdf]

Tailoring Electronic and Optical Properties of hBN/InTe and hBN/GaTe Heterostructures Through Biaxial Strain Engineering: Supplementary Information

A. Šolajić[1,*] and J. Pešić[1,2]

[1]Laboratory for 2D materials, Center for Solid State Physics and New Materials, Institute of Physics Belgrade, University of Belgrade, Pregrevica 118, 11080 Belgrade, Serbia
[2]Chair of Physics, Department Physics, Mechanics and Electrical Engineering, Montanuniversität Leoben, 8700 Leoben, Austria
[*]solajic@ipb.ac.rs

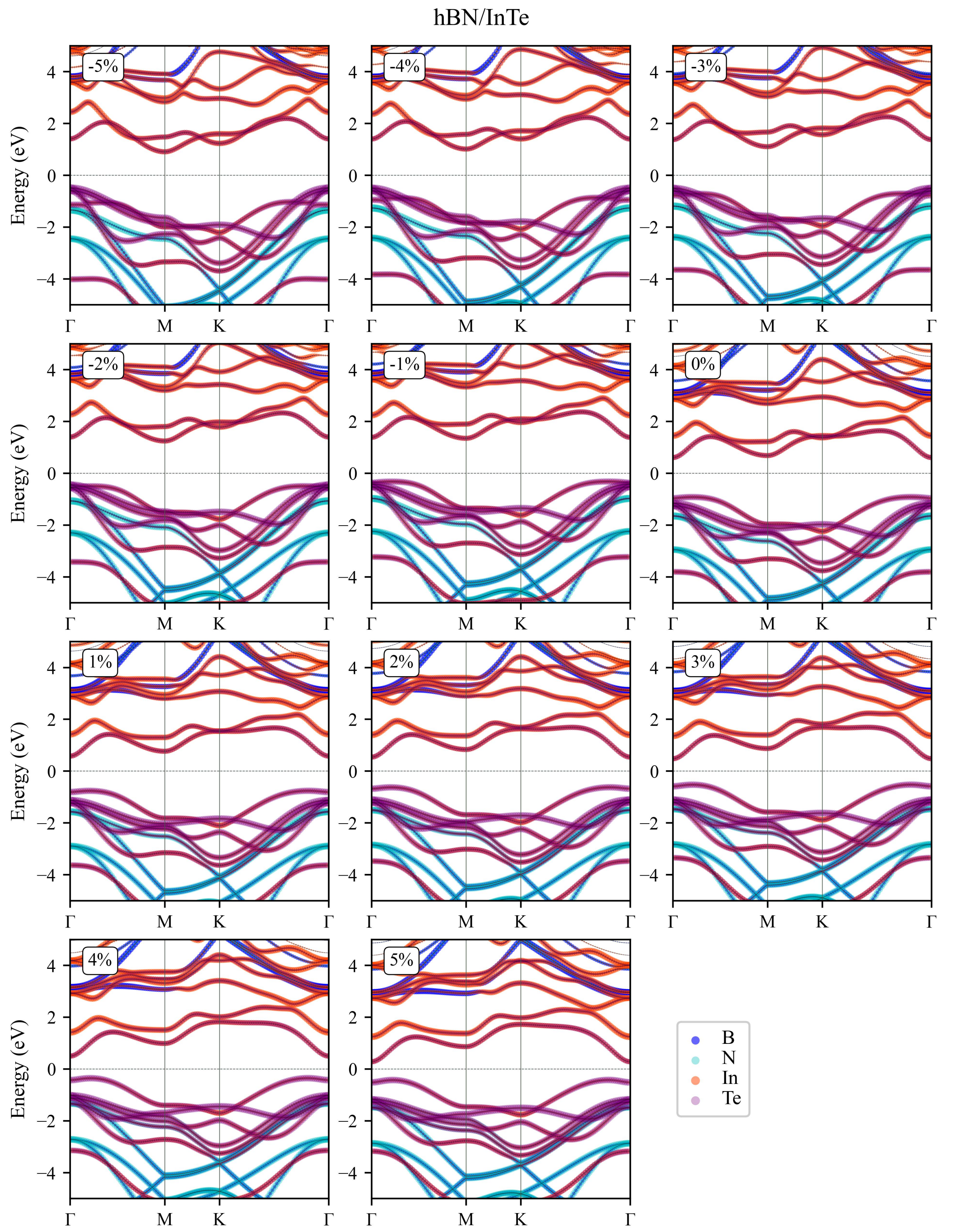

Figure S1. Band structure of hBN/InTe HS for different values of applied strain. The width of the lines is proportional to the contribution of different atoms/states. The line width is proportional to the magnitude of projections of wavefunctions over atomic orbitals. The contributions from different atomic orbitals are presented in different colours, as shown in the legend.

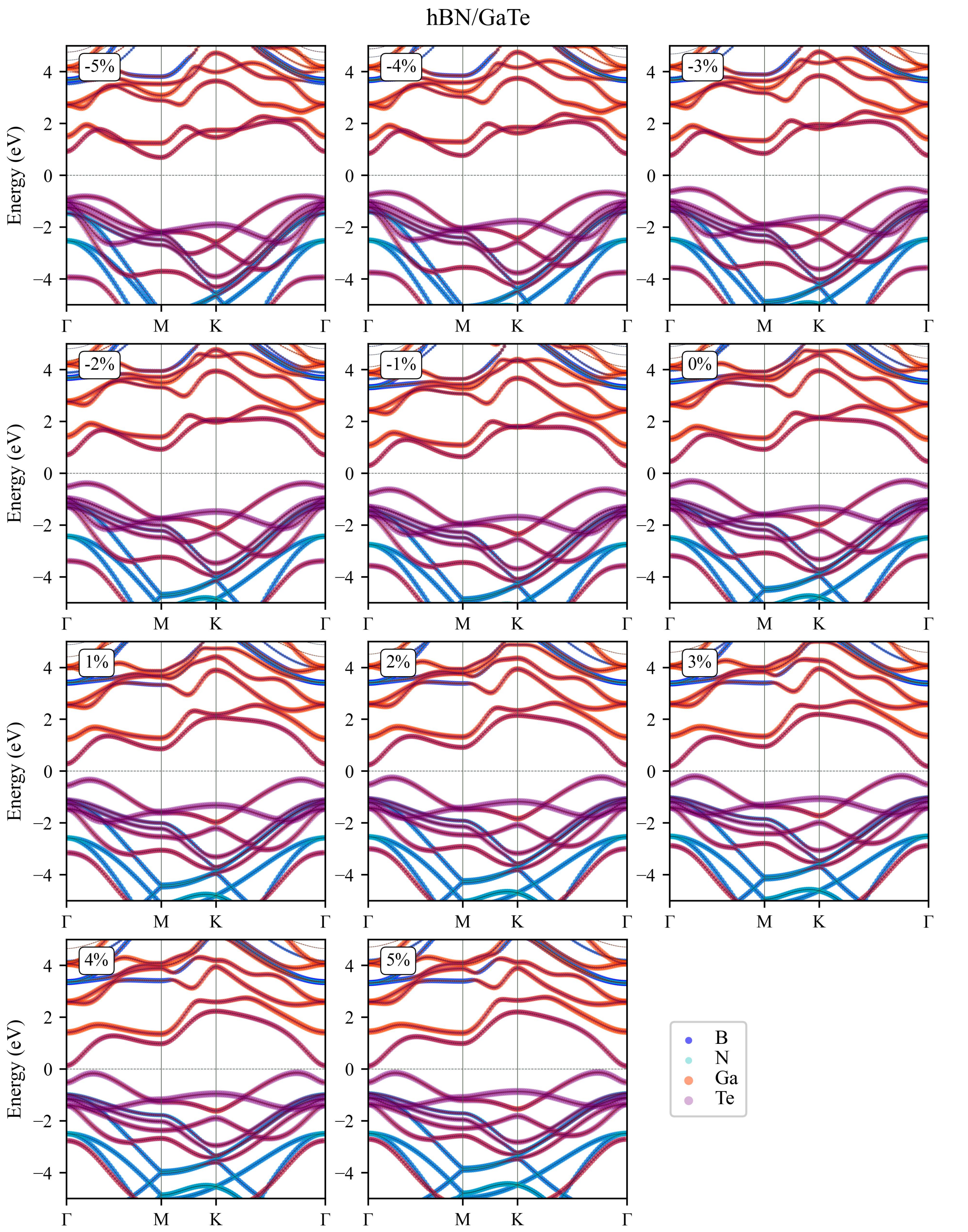

Figure S2. Band structure of hBN/GaTe HS for different values of applied strain. The width of the lines is proportional to the contribution of different atoms/states. The line width is proportional to the magnitude of projections of wavefunctions over atomic orbitals. The contributions from different atomic orbitals are presented in different colours, as shown in the legend.

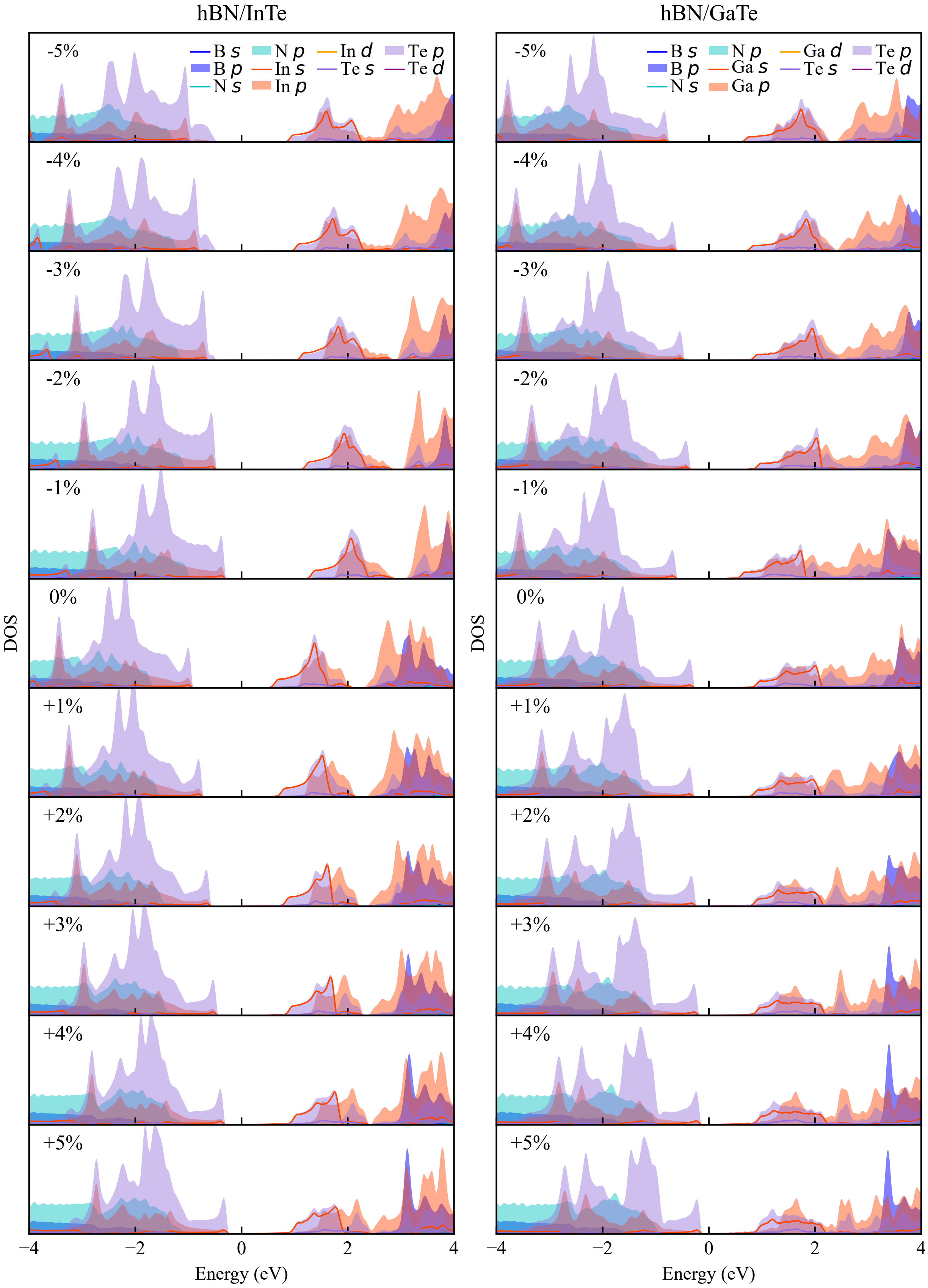

Figure S3. Projected density of states of hBN/InTe HS on the left, and hBN/GaTe HS on the right, for each strength of strain applied. Contributions from different atoms and states are represented with colours as in legend.
